# Human in vivo evidence of associations between herpes simplex virus and cerebral amyloid-beta load in normal aging

**DOI:** 10.1186/s13195-024-01437-4

**Published:** 2024-04-03

**Authors:** Jose L. Cantero, Mercedes Atienza, Isabel Sastre, María Jesús Bullido

**Affiliations:** 1 Laboratory of Functional Neuroscience, Pablo de Olavide University, Ctra. de Utrera Km 1, Seville, 41013 Spain; 2 Centro de Investigación Biomédica en Red de Enfermedades Neurodegenerativas (CIBERNED), Instituto de Salud Carlos III, Madrid, Spain; 3 Instituto de Investigación Sanitaria del Hospital Universitario La Paz, IdiPAZ (Hospital Universitario La Paz – Universidad Autónoma de Madrid), Madrid, Spain; 4 Centro de Biología Molecular “Severo Ochoa” (C.S.I.C.-U.A.M.), Universidad Autónoma de Madrid, Madrid, Spain

**Keywords:** Herpes simplex virus, Aging, Amyloid PET, Neurodegeneration, Alzheimer’s disease, Amyloid-beta plaques, APOE4

## Abstract

**Background:**

Mounting data suggests that herpes simplex virus type 1 (HSV-1) is involved in the pathogenesis of AD, possibly instigating amyloid-beta (Aβ) accumulation decades before the onset of clinical symptoms. However, human in vivo evidence linking HSV-1 infection to AD pathology is lacking in normal aging, which may contribute to the elucidation of the role of HSV-1 infection as a potential AD risk factor.

**Methods:**

To shed light into this question, serum anti-HSV IgG levels were correlated with ^18^F-Florbetaben-PET binding to Aβ deposits and blood markers of neurodegeneration (pTau181 and neurofilament light chain) in cognitively normal older adults. Additionally, we investigated whether associations between anti-HSV IgG and AD markers were more evident in APOE4 carriers.

**Results:**

We showed that increased anti-HSV IgG levels are associated with higher Aβ load in fronto-temporal regions of cognitively normal older adults. Remarkably, these cortical regions exhibited abnormal patterns of resting state-functional connectivity (rs-FC) only in those individuals showing the highest levels of anti-HSV IgG. We further found that positive relationships between anti-HSV IgG levels and Aβ load, particularly in the anterior cingulate cortex, are moderated by the APOE4 genotype, the strongest genetic risk factor for AD. Importantly, anti-HSV IgG levels were unrelated to either subclinical cognitive deficits or to blood markers of neurodegeneration.

**Conclusions:**

All together, these results suggest that HSV infection is selectively related to cortical Aβ deposition in normal aging, supporting the inclusion of cognitively normal older adults in prospective trials of antimicrobial therapy aimed at decreasing the AD risk in the aging population.

## Background

Alzheimer’s disease (AD) is a multifactorial neurodegenerative disorder originated by a mixture of etiopathogenic mechanisms, which likely arise from complex interactions among genetics, lifestyle and environmental risk factors. Substantial evidence suggests that the aggregation of amyloid-beta (Aβ) isoforms into senile plaques is the earliest recognizable pathological event in AD. These first Aβ deposits precede overt clinical symptoms by decades [1, 2] and they supposedly initiate the pathogenic cascade that ultimately leads to cognitive decline [3]. Identifying risk factors for Aβ deposition in asymptomatic older adults is of paramount importance to understand AD etiology and to ameliorate AD-related brain lesions before cognitive decline appears.

Persistent viral infections have been suggested to play a role in AD pathogenesis, particularly the herpes simplex virus type 1 (HSV-1) [4–7], a ubiquitous neurotropic human pathogen infecting over 67% of the world’s population [8]. The HSV-1 virus first infects oral and nasal surface epithelia and then travels through retrograde axonal transport to the trigeminal ganglia to establish a latent infection, pointing to ganglionic sensory neurons as a lifelong reservoir of recurrent infections [9]. A variety of factors, such as stress, inflammation, immunosuppression, fever, hormone imbalance, exposure to ultraviolet light, or injury to the tissues innervated by latently-infected neurons, are able to engage the latent virus in productive replication, affecting HSV-1-infected neurons or surrounding, non-neuronal cells [10].

Convergent research has led to the hypothesis that cumulative effects of recurrent HSV brain infections may increase AD pathology [11–13]. However, there is lack of human in vivo evidence linking HSV to hallmarks of AD pathology in normal aging, which may contribute to shed light on the role of HSV infection as a potential AD risk factor before the onset of cognitive deficits. To specifically address this question, we have investigated whether increased serum levels of anti-HSV IgG in cognitively normal older adults is associated with increased cortical Aβ load, as revealed by amyloid PET imaging, and/or with blood markers of neurodegeneration, as shown by neurofilament light chain (NfL) and tau phosphorylated at threonine-181 (pTau-181) levels. We expected a positive association between anti-HSV IgG levels and cortical Aβ load based on evidence that HSV infection leads to altered processing of the amyloid precursor protein (APP) [14] and increases enzymes responsible for Aβ formation [15].

Postmortem studies have revealed that the majority of Aβ plaques located in fronto-temporal cortices of AD patients and non-demented older individuals contained HSV-1 DNA [16]. Consequently, associations between anti-HSV IgG and binding of the amyloid PET tracer to Aβ deposits are expected to be mostly restricted to frontal and temporal cortical regions, which may also exhibit altered patterns of functional connectivity in individuals showing the highest levels of anti-HSV IgG. We did not expect associations between anti-HSV IgG levels and blood markers of neurodegeneration, since cognitive deficits are mostly induced by tau pathology occurring subsequently to Aβ deposition in confirmed AD patients [17, 18].

Gene products of the HSV-1 life cycle have been shown to interact with the apolipoprotein E allele 4 (APOE4), the strongest genetic risk factor in the sporadic form of AD, to instigate both viral infectivity and increase AD risk [19–21]. Based on these findings, we hypothesized that associations between serum levels of anti-HSV IgG and cortical Aβ load would be more evident in older individuals carrying the APOE4 genotype.

## Methods

### Participants

Sixty-five cognitively normal older adults participated in the study (66.8 ± 5.5 years; age range: 54–76 years; 44 females). They were recruited from senior citizen’s associations, health-screening programs, and hospital outpatient services. All of them underwent neurological and neuropsychological assessment to discard the presence of cognitive impairment and/or dementia. Individuals with medical conditions affecting brain structure or function (e.g., cerebrovascular disease, epilepsy, head trauma, history of neurodevelopmental disorders, mental retardation, alcohol abuse, hydrocephalus, and/or intracranial mass) were not included in the study. Participants met the following criteria: (i) normal global cognitive status in the Mini-Mental State Examination (scores ≥ 26); (ii) normal cognitive performance in the neuropsychological tests relative to appropriate reference values for age and education level; (iii) global score of 0 (no dementia) in the Clinical Dementia Rating; (iv) functional independence as assessed by the Spanish version of the Interview for Deterioration in Daily Living Activities [22]; (v) scores ≤ 5 (no depression) in the short form of the Geriatric Depression Scale [23]; and (vi) not be taking medications that affected cognition, sleep, renal and/or hepatic function. All participants gave informed consent to the experimental protocol approved by the Ethical Committee for Clinical Research of the Junta de Andalucía according to the principles outlined in the Declaration of Helsinki.

### Neuropsychological assessment

Participants were administered with a comprehensive battery of neuropsychological tests to assess memory, language, attention, processing speed, and executive function skills such as working memory, inhibition, flexibility and planning. Cognitive tests included the Spanish version of the memory binding test (MBT) [24], the face-name associative memory exam (S-FNAME) [25], the D2 test, the letter-number sequencing and the digit span subtests of the Wechsler Adult Intelligence Scale-III, the spatial span subtest of the Wechsler Memory Scale-III, the short form of the Boston naming test (BNT), the semantic and phonological fluency tests based on the “Animal” and letter “P” naming tasks, the symbol digit modalities test, the two forms of the trail making test (TMT-A and TMT-B), and the tower of London (TL). All scores were z transformed. Inverse z-values were used when higher scores corresponded to worse performance. We further computed the Spearman’s ‘g’ factor as an index of global cognitive function. This analysis was done with R Statistical Software v3.0.1 (R Foundation for Statistical Computing, Vienna, Austria) using the *prcomp* function.

### Serological determination of HSV infection status

Fasting blood samples were collected at 9:00–10:00 AM in all participants, immediately processed, and stored at -80ºC until analysis. Serological evaluation of HSV infections were assessed with Enzygnost® enzyme-linked immunosorbent assays (Siemens Healthcare, Marburg, Germany), following the manufacturer’s instructions. These assays allow qualitative detection and quantitative determination of human IgG (respectively IgM) antibodies to HSV in serum, showing a sensitivity of 93% and a specificity of 100% for anti-HSV IgG, and a sensitivity of 92% and a specificity of 93% for anti-HSV IgM, according to the manufacturer. However, they do not distinguish between HSV-1 and HSV-2.

### Blood markers of neurodegeneration

Serum NfL and plasma pTau-181 levels were measured on the ultra-sensitive single-molecule array (Simoa®) SR-X platform (Quanterix, MA, USA) following the manufacturer’s instructions. The Simoa® NF-light Advantage v2 (Cat. Nº 104,073) and pTau-181 Advantage v2.1 (Cat. Nº 104,111) assays were purchased from Quanterix. These assays measure serum NfL and plasma pTau-181 levels with a detection limit of 0.141 and 1.04 pg/ml, respectively. Two quality control samples were run on each plate for each analyte. Determinations were run in duplicates, and the average of the two measurements (pg/ml) was used for statistical analysis. Samples with coefficients of variation above 20% were repeated.

### APOE genotyping

Genomic DNA was isolated from blood using a standard salting-out protocol [26]. APOE polymorphisms were determined with pre-designed TaqMan® single nucleotide polymorphism genotyping assays (Applied Biosystem™, Thermo Fisher Scientific).

### Acquisition and preprocessing of amyloid PET data

Participants were injected with 300 MBq of 18 F-Florbetaben (FBB, NeuraCeq™, Curium Pharma) 90 min before image acquisition in a Philips Gemini 16 PET/CT scanner (Philips, Best, Netherlands). They underwent a 20-min FBB-PET scan in dynamic mode consisting of four frames of 5 min each. Each frame was inspected for excessive motion. As no excessive head motion was detected in FBB images, the four frames were averaged to create a single static FBB image used for quantitative analysis. Amyloid PET scans were corrected for radioactive decay, dead time, attenuation, and scatter, and they were reconstructed iteratively with an isotropic voxel resolution of 2 mm^3^.

Partial volume correction (PVC) of FBB images was performed with PetSurfer (https://surfer.nmr.mgh.harvard.edu/fswiki/PetSurfer) using the geometric transfer matrix-derived region-based voxel-wise method and assuming a uniform 6 mm point spread function [27]. Briefly, FBB images were first co-registered to T1 scans. Next, PVC-cortical FBB images were transformed into standardized uptake value ratio (SUVR) using the mean PVC uptake of the cerebellar GM as reference region. Resulting PVC-FBB cortical-to-cerebellum SUVR images were mapped into individual cortical surfaces and they were finally smoothed with non-linear spherical wavelet-based de-noising schemes [28].

### Acquisition and preprocessing of MRI data

Brain images were acquired on a 3T Philips Ingenia MRI scanner using a 32-channel receive-only radio-frequency (RF) head coil and a transmit RF body coil (Philips, Best, Netherlands). The following MRI sequences were acquired in the same session: (i) 3D T1-weighted (T1w) magnetization prepared rapid gradient echo (MPRAGE) in the sagittal plane: repetition time (TR)/echo time (TE) = 2600 ms/4.7 ms, flip angle (FA) = 9°, acquisition matrix = 384 × 384, voxel resolution in acquisition = 0.65 mm^3^ isotropic, resulting in 282 slices without gap between adjacent slices; and (ii) T2w Fast Field Echo images using a blood-oxygen-level-dependent (BOLD) sensitive single-shot echo-planar imaging (EPI) sequence in the axial plane: TR/TE: 2000 ms/30 ms, FA = 80°, acquisition matrix = 80 × 80 mm, voxel resolution in acquisition = 3 mm^3^ isotropic, resulting in 35 slices acquired in posterior to anterior phase-encoding direction with 1 mm of gap between adjacent slices. Pulse and respiratory rates were simultaneously recorded using the scanner’s built-in pulse oximeter placed on the left-hand index finger and a pneumatic respiratory belt strapped around the upper abdomen, respectively. Before starting the acquisition of the EPI sequence, participants were asked to remain still and keep their eyes closed without falling sleep. We acquired 250 EPI scans preceded by 4 dummy volumes to allow time for equilibrium in the spin excitation. To facilitate optimal B1 shimming, a B1 calibration scan was applied before starting the EPI sequence. Brain images were visually examined after each MRI sequence; they were repeated if evident artifacts were identified. All participants underwent the same MRI protocol in the same scanner at the research MRI facility of Pablo de Olavide University.

T1w scans were preprocessed using Freesurfer v6.0 (https://surfer.nmr.mgh.harvard.edu/). The Freesurfer’s pipeline included brain extraction, automated tissue segmentation, generation of white matter (WM) and pial surfaces, correction of surface topology and inflation, co-registration, and projection of cortical surfaces to a sphere for the purpose of establishing a surface-based coordinate system [29]. Pial surface misplacements and erroneous WM segmentation were manually corrected on a slice-by-slice basis by one experienced technician. All processing steps were visually checked for quality assurance.

The resting state-fMRI (rs-fMRI) data were preprocessed using AFNI functions (https://afni.nimh.nih.gov/afni), version AFNI_20.3.01. For each participant, high-frequency spikes were eliminated (*3dDespike*), time-locked cardiac (measured by pulse oximeter) and respiratory motion artifacts on brain BOLD signals were minimized using RETROICOR [30], time differences in slice-acquisition were corrected (*3dTshift*), EPI scans were aligned using the first volume as reference and rigid body motion correction (*3dVolreg*), and aligned EPI scans were co-registered to their corresponding T1w volumes (*align_epi_anat.py*; cost function: lpc + ZZ). Dynamics were removed provided that more than 5% of voxels exhibited signal intensities that deviated from the median absolute deviation of time series (*3dToutcount*), and/or when the Euclidean norm (*enorm*) threshold exceeded 0.3 mm in head motion. None of the participants showed more than 20% of artifactual dynamics after applying censoring. Simultaneous regression was further applied to minimize the impact of non-neuronal fluctuations on the rs-fMRI signal (*3dTproject*). Nuisance regressors included: (i) six head motion parameters (3 translational and 3 rotational) along with their first-order derivatives, (ii) time series of mean total WM/CSF signal intensity, and (iii) cardiac and respiratory fluctuations plus their derivatives to mitigate effects of extracerebral physiological artifacts on brain BOLD signals.

Preprocessed rs-fMRI scans were projected onto the 5th order icosahedral tesselation of the average cortical surface. Seeds for rs-FC analyses were obtained from cortical regions showing significant correlations between anti-HSV IgG levels and FBB binding. Surface-based rs-FC seed to whole cortex maps were computed using the Fisher’s z-transform of the corresponding Pearson’s correlation coefficients.

### Statistical analysis

One-way ANOVAs were performed to analyze anti-HSV IgG differences as a function of sex or APOE4 genotype. Multiple linear regression analyses were further performed to evaluate associations of anti-HSV IgG levels with age, NfL and pTau-181, separately. These analyses were adjusted by age, sex, and APOE4 as long as they were not regressors of interest.

We next assessed associations between anti-HSV IgG levels and cognitive function. Five cognitive domains were analyzed: memory, language, attention, processing speed, and executive function. To improve normality and alleviate heteroscedasticity, composite Z-scores for each cognitive domain were Yeo-Johnson transformed [31]. A four-step mixed effects model including random intercepts across participants was applied to reduce de variance of fixed effect estimates [32]. The covariates were included in the first step, whereas the main effects (i.e., cognitive domain, anti-HSV IgG levels, anti-HSV IgG-related variations in cortical FBB binding, age, sex, and APOE4) as well as the two- three- and four-way interactions were sequentially added in subsequent steps. We finally applied ANOVAs to compare the different models. These analyses were performed with R Statistical Software v3.0.1 (R Foundation for Statistical Computing, Vienna, Austria).

Vertex-wise multiple linear regression analysis was conducted on cortical surfaces to assess associations between anti-HSV IgG levels and variations in FBB binding. FBB cortical maps and anti-HSV IgG levels were previously Box-Cox transformed to improve normality and alleviate heteroscedasticity [33]. Significant cortical regions resulting from this analysis were used as seeds to evaluate whether positive rs-FC patterns were indeed moderated by anti-HSV IgG levels. All statistical models were adjusted for age, sex, and APOE4. We next assessed whether APOE4 moderated associations between anti-HSV IgG levels and cortical FBB binding. This statistical model was adjusted for age and sex.

Cortical surface-based analyses were performed using the SurfStat package (https://www.math.mcgill.ca/keith/surfstat/). Results were corrected for multiple comparisons using a validated hierarchical statistical model [34]. This procedure first controls the family-wise error rate in significant clusters over smoothed statistical maps applying random field theory; and it next controls the false discovery rate in vertices of significant clusters over unsmoothed statistical maps. The anatomical location of clusters that survived corrections was identified by the location of each cluster’s peak vertex on the Desikan-Killiany atlas [35]. After the inferential evidence of a significant effect, the standardized local effect size (f^2^_local_ or rho_local_) was further computed within the context of a multivariate regression model [36]. To establish the precision of effect sizes, 95% confidence intervals (CI_95%_) were estimated with the function *bootci* implemented in Matlab (10.000 bootstrap samples), using the normal approximated interval with bootstrapped bias and standard error.

Bayesian linear regression analysis was applied to peak vertices of significant cortical cluster using JASP, version 0.12.2 (https://jasp-stats.org/). The Bayesian approach allowed us, on the one hand, to interpret results in terms of the magnitude of evidence in favor of the alternative hypothesis as opposed to the null hypothesis and, on the other, to overcome the problem of multiple comparisons across statistical contrasts. Bayesian linear regression analyses were based on the Jeffreys-Zellner-Siow prior using an r scale of 0.354 [37]. The strength of the Bayes factor for the model including all covariates of no interest (null model) was compared with the model including the predictor of interest (experimental model) (BF_10_). BF_10_ values were interpreted using the classification scheme proposed by Lee and Wagenmakers [38]. We only reported results that met all the following criteria: (i) the *p* value yielded by the frequentist approach was < 0.05 after correction for multiple comparisons, (ii) the standardized local effect size was at least moderate and the CI_95%_ did not contain zero, and (iii) the evidence in favor of the alternative hypothesis was at least moderate (BF_10_ ≥ 3).

## Results

### Anti-HSV levels and clinical characteristics

Table 1 shows demographics and clinical characteristics of the sample. Only five out of 65 participants (7.7%) were found positive for HSV IgM, precluding the possibility to study them as a separate group. None of the participants showed IgG- IgM + status. Anti-HSV IgG levels were not affected by either sex or APOE4 genotype, as revealed by one-way ANOVAs. Neither age nor cognitive performance (either global cognition or each cognitive domain separately) showed significant correlations with anti-HSV IgG levels after adjustment for covariates.

**Table 1:** Demographics and clinical characteristics of the sample

Age (years)	66.8 ± 5.5
Sex (F/M)	44/21
Education years	12.3 ± 5.1
ApoE4 (yes/no)	13/52
MMSE	29.0 ± 1.2
Memory Binding Test	
Total free recall Total paired recall Total delayed free recall Total delayed paired recall	15.3 ± 4.5 24.5 ± 4.3 16.1 ± 4.9 23.7 ± 4.8
S-FNAME	31.6 ± 15.2
Boston Naming Test	11.9 ± 2.1
Phonological fluency	15.6 ± 4.4
Semantic fluency	21.8 ± 17.9
Trail Making Test-A	47.7 ± 22.3
Trail Making Test-B	123.0 ± 70.2
Tower of London	324.3 ± 113.7
D2	363.3 ± 97.1
Letter-number sequencing	9.0 ± 2.5
Digit span	14.3 ± 3.3
Symbol digit modalities test	37.7 ± 12.1
Anti-HSV-1 IgG (titer)	95804.6 ± 60821.8
Anti-HSV-1 IgM (positive)	5
NfL (pg/ml)	14.4 ± 7.5
pTau-181 (pg/ml)	23.5 ± 9.6

Results are expressed as mean ± standard deviation (SD), unless otherwise stated. F/M: females/males; MMSE: Mini Mental State Examination; S-FNAME: Spanish validation of the Face Name Associative Memory Exam. NfL: neurofilament light chain

### Associations between anti-HSV IgG levels and AD markers

**Fig. 1:**
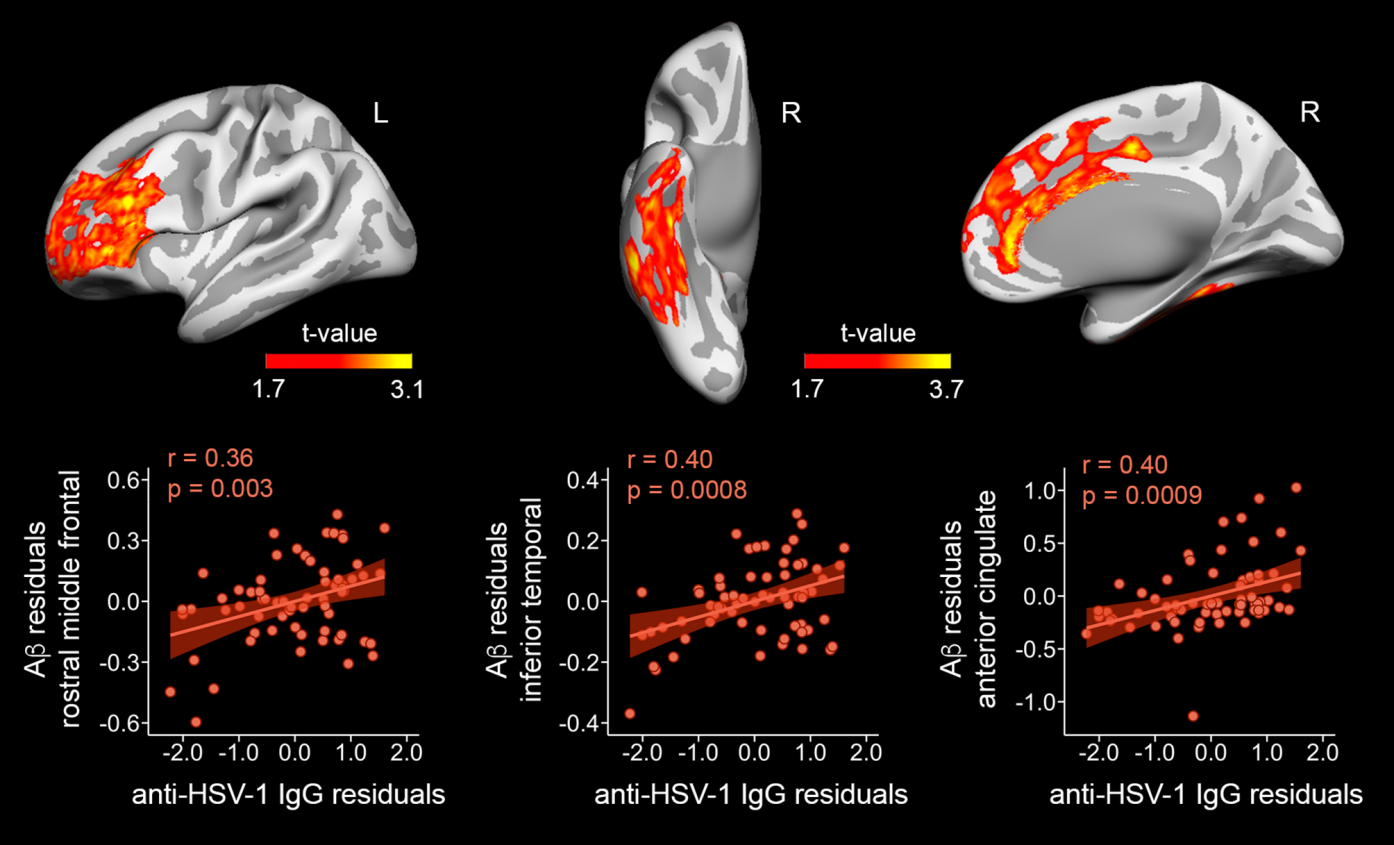
Association between anti-HSV IgG levels and cortical Florbetaben (FBB) binding. Significant results are projected on cortical surfaces (upper panel). Scatter plots (bottom panel) show the relationship of anti-HSV IgG levels with the peak vertex of cortical FBB binding after adjustment by age, sex, and APOE4

**Fig. 2:**
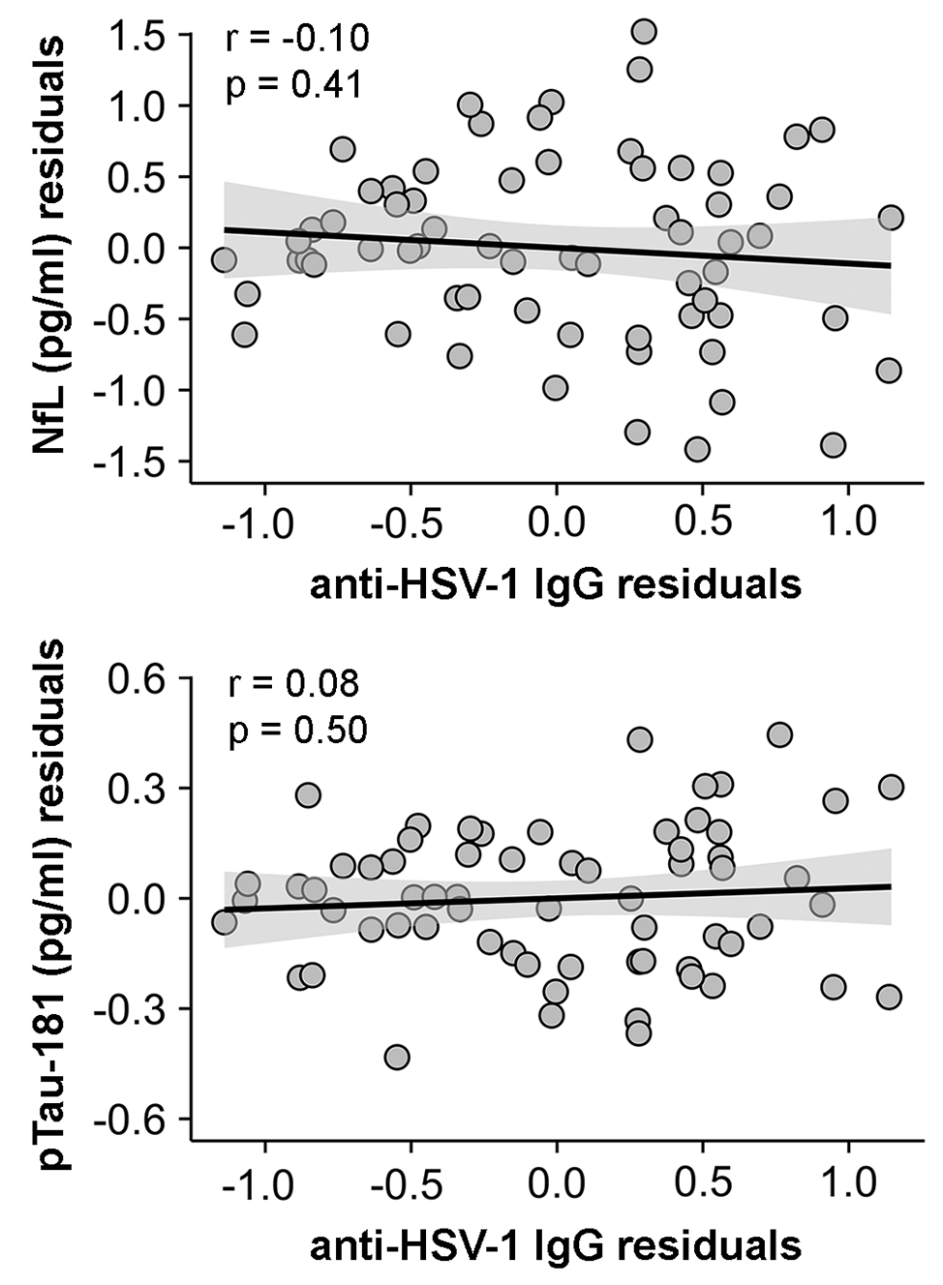
Association between anti-HSV IgG levels and blood markers of neurodegeneration. Scatter plots show the relationship of anti-HSV IgG levels with neurofilament light chain (NfL) (upper panel) and pTau-181 (bottom panel) after adjustment by age, sex, and APOE4

Surface-based regression analysis revealed that anti-HSV IgG levels were positively correlated with FBB binding in different regions of the cortical mantle. Results are illustrated in Fig. 1 and summarized in Table 2. Anti-HSV IgG levels were positively correlated with increased Aβ load in rostral middle frontal regions of the left hemisphere, and anterior cingulate and inferior temporal regions of the right hemisphere. Effect sizes appeared moderate in all cases and Bayesian linear regression analyses showed strong evidence in favor of the alternative hypothesis for the left rostral middle frontal regions and very strong evidence for the right anterior cingulate and inferior temporal regions (Table 2). No significant relationships were found between anti-HSV IgG levels and blood markers of neurodegeneration (neither NfL nor pTau-181) (Fig. 2).

**Table 2:** Cortical regions showing significant associations between anti-HSV-1 IgG levels and Aβ load

Positive correlations	Extent of change (mm^2^)	MNI	R^2^	F_11,53_	${f}_{local}^{2}$	CI_95%_	BF_10_
L rostral middle frontal (*p*_*cluster*_= 0.0002)	6025	-42 35 22	0.13	9.8	0.16^M^	0.008–0.52	14^S^
R anterior cingulate (*p*_*cluster*_= 0.00002)	3219	4 20 20	0.18	13.6	0.19^M^	0.07–0.44	35^VS^
R inferior temporal (*p*_*cluster*_= 0.008)	2981	57 − 39 -22	0.18	13.7	0.19^M^	0.02–0.61	39^VS^

MNI coordinates are in MNI152 space. Local effect size (*f*^2^_local_): ^M^ moderate. CI_95%_: 95% confidence interval. BF_10_: Bayes factor derived from Bayesian linear regression analyses. The superscript of the BF_10_ indicates the qualitative interpretation of the magnitude of evidence in favor of the alternative hypothesis as opposed to the null hypothesis: ^S^ strong; ^VS^ very strong. L: left; R: right

### Effect of anti-HSV IgG levels on rs-FC

**Fig. 3:**
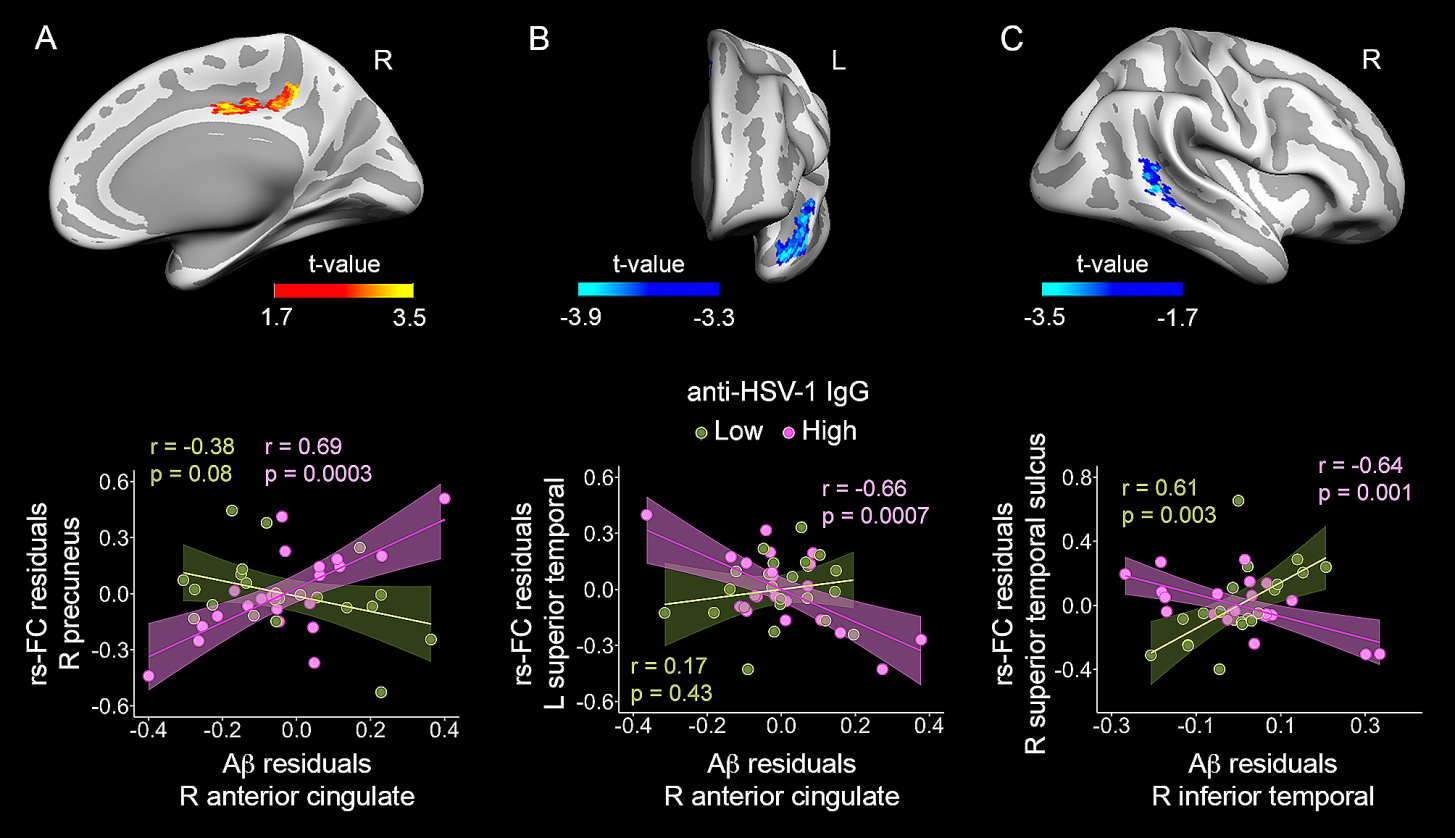
Moderating role of anti-HSV IgG levels in the relationship between regional FBB binding and rs-FC patterns. Positive and negative partial correlations adjusted by age, sex and APOE4 are displayed in warm and cool colors, respectively. **(A)** Abnormal pattern of rs-FC using the right anterior cingulate as FC seed (upper panel). Only participants showing the highest anti-HSV IgG levels (pink) exhibited significant positive associations between Aβ load in the right anterior cingulate and rs-FC of the right anterior cingulate with the right precuneus (bottom panel). **(B)** Abnormal pattern of rs-FC using the right anterior cingulate as FC seed (upper panel). Only participants showing the highest anti-HSV IgG levels (pink) exhibited significant negative associations between Aβ load in the right anterior cingulate and rs-FC of the right anterior cingulate with the left superior temporal cortex (bottom panel). **(C)** Abnormal pattern of rs-FC using the right inferior temporal as FC seed (upper panel). Only participants showing the highest anti-HSV IgG levels (pink) exhibited significant negative associations between Aβ load in the right inferior temporal cortex and rs-FC of the right inferior temporal cortex with the right superior temporal sulcus (bottom panel). This pattern of rs-FC appeared significantly opposite in older adults showing the lowest anti-HSV IgG levels (green). Right: right, L: left

By using as seeds the clusters resulting from significant associations between anti-HSV IgG levels and FBB binding, we next assessed whether the relationship of Aβ load of these clusters with their patterns of rs-FC differed as a function of anti-HSV IgG levels. Results are shown in Fig. 3 and summarized in Table 3. Multiple regression analyses revealed a moderating role of anti-HSV IgG levels for the association between Aβ load in the right anterior cingulate and the strength of rs-FC of this region with the right precuneus (Fig. 3A, upper panel). *Post hoc* analyses showed that only those participants having 1 standard deviation (SD) above the mean of anti-HSV IgG levels exhibited a significant positive association between these variables (Fig. 3A, bottom panel).

**Table 3:** Effects of the interaction anti-HSV-1 IgG levels × Aβ load on rs-FC patterns

FC seed Peak location of significant result	Extent of change (mm^2^)	MNI	R^2^	F_8,58_	${rho}_{local}$	CI_95%_	BF_10_
**R anterior cingulate (FC seed)**							
L superior temporal (*p*_*cluster*_= 0.005)	289	-48 14 − 13	0.20	15.6	0.95^L^	-1.49 – -0.57	128^E^
R precuneus (*p*_*cluster*_= 0.02)	499	8–41 45	0.22	18.0	0.92^L^	0.56–1.40	9^M^
***R inferior temporal gyrus (FC seed)***							
R superior temporal (*p*_*cluster*_= 0.01)	425	56 − 41 3	0.16	12.1	0.84^L^	-1.31 – -0.56	7^M^

MNI coordinates are in MNI152 space. Local effect size (*rho*_local_): ^L^ large. CI_95%_: 95% confidence interval. BF_10_: Bayes factor derived from Bayesian linear regression analyses. The superscript of the BF_10_ indicates the qualitative interpretation of the magnitude of evidence in favor of the alternative hypothesis as opposed to the null hypothesis: ^M^ moderate; ^E^ extreme. L: left; R: right

The moderating role of anti-HSV IgG levels was also evident for the association between Aβ load in the right anterior cingulate and the rs-FC of this region with the left superior temporal gyrus (Fig. 3B, upper panel). This association was negative for participants with the highest anti-HSV IgG levels (Fig. 3B, bottom panel). A similar result was found for the association between Aβ load in the right inferior temporal gyrus and the rs-FC of this region with the right superior temporal sulcus (Fig. 3C, upper panel). In this case, both participants showing the highest and the lowest anti-HSV IgG levels exhibited significant associations in opposite directions (Fig. 3C, bottom panel).

### Effect of APOE4 genotype on the relationship between anti-HSV IgG levels and cortical Aβ load

**Fig. 4:**
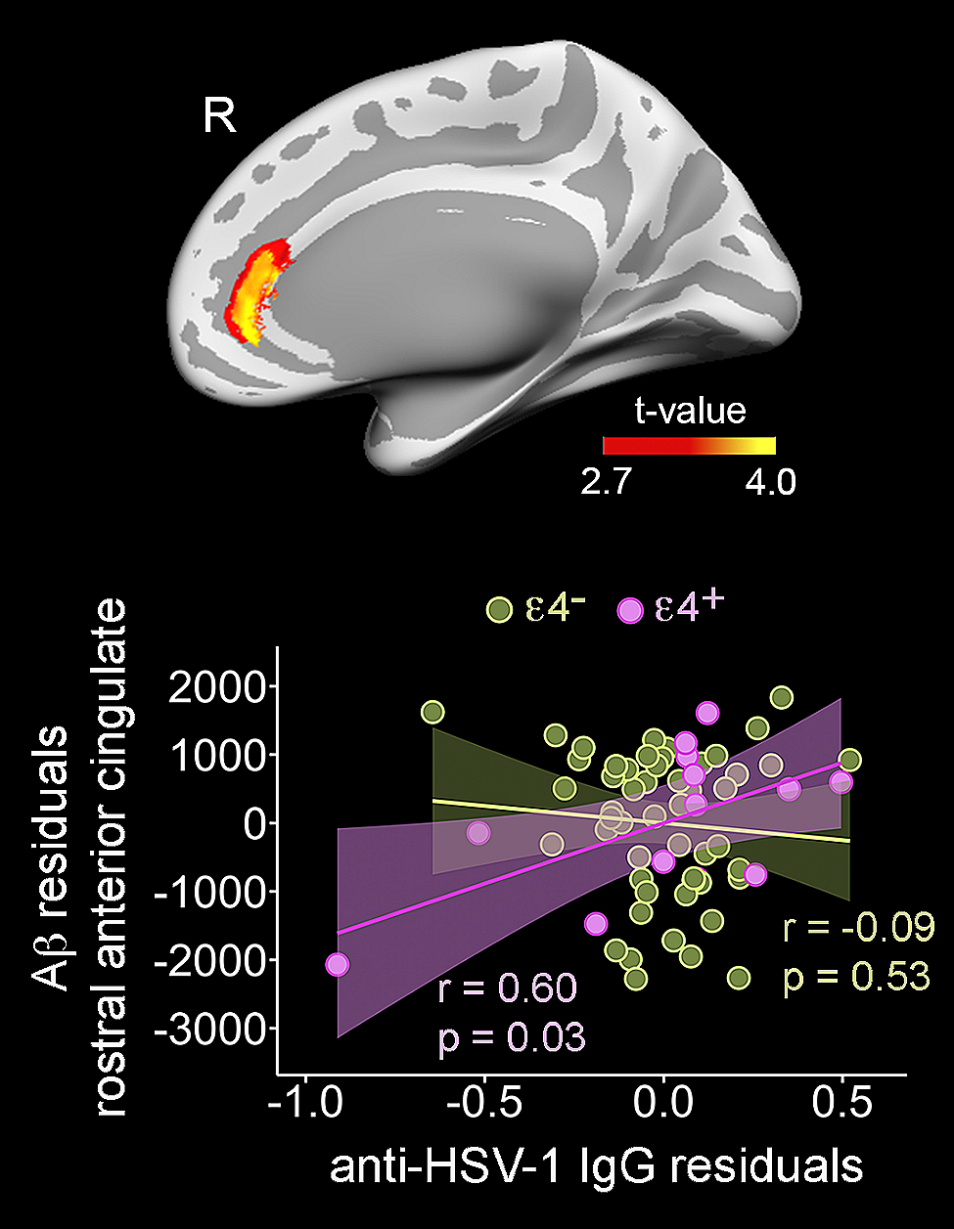
Moderating effect of APOE4 on the relationship between anti-HSV IgG levels and cortical Florbetaben (FBB) binding. Results of the regression model is projected on the cortical surfaces (upper panel). The scatter plot (bottom panel) shows the partial correlation between anti-HSV IgG levels and Aβ load in the rostral anterior cingulate adjusted by age and sex in APOE4 carriers (pink) and noncarriers (green). Right: right

Finally, we found that the APOE4 genotype moderated the association between anti-HSV IgG levels and FBB binding in the right rostral anterior cingulate (F_11,53_ = 16.4; *p* = 0.0006, delta_local_ = 2.17, CI_95%_ [0.11–5.33], BF_10_ = 37.4) (Fig. 4, upper panel). *Post hoc* analyses indicated that only APOE4 carriers showed a significant positive association between anti-HSV IgG levels and Aβ load in the rostral anterior cingulate cortex of the right hemisphere after adjustment by age and sex (*r* = 0.60, *p* = 0.03) (Fig. 4, bottom panel).

## Discussion

Growing evidence suggests that HSV-1 is involved in the pathogenesis of AD, probably contributing to neuroinflammation decades before the onset of clinical symptoms. Despite this, no study to date has provided human in vivo evidence linking HSV infection to AD pathology in normal aging, a phase of life in which HSV reactivation in the brain increases in parallel with a decrease in adaptive immunity and a higher permeability of the blood-brain barrier (BBB). In line with our initial hypothesis, we showed a positive association between anti-HSV IgG levels and Aβ load in fronto-temporal regions of cognitively normal older adults. Interestingly, cortical regions affected by this relationship also exhibited abnormal patterns of resting-state hemodynamic synchronization with the precuneus and temporal lobe, two cortical regions largely affected by AD pathology from the earliest stages. We further found that associations between HSV infection and Aβ load, particularly in the anterior cingulate cortex, were moderated by the APOE4 genotype. Importantly, anti-HSV IgG levels were unrelated to either subclinical cognitive deficits or to blood markers of neurodegeneration, suggesting that anti-HSV IgG was selectively associated with cortical Aβ aggregation in normal aging. To our knowledge, these results provide the first evidence that HSV infection relates to cerebral Aβ deposition in normal aging, supporting the inclusion of cognitively normal older adults in prospective trials of antimicrobial therapy aimed at decreasing the AD risk in the aging population.

The relationship between aging-related deregulations of the immune response, HSV infection, and cerebral Aβ burden is extremely complex. Each of these elements separately may alter the integrity of the BBB, facilitating the transport of HSV into the brain parenchyma, and ultimately exacerbating Aβ aggregation and neuroinflammation [39]. Alternatively (or reciprocally), the increased production of pro-inflammatory cytokines and plasma cells with aging [40, 41] may also lead to an increased production of autoantibodies in body fluids aimed at maintaining adaptive immunity through B cell dysfunction [42]. From this perspective, increased serum levels of anti-HSV IgG may be interpreted as a mere epiphenomenon of inflammaging, which may contribute to accelerated immunosenescence and HSV reactivation [43, 44], and incidentally relate to cortical Aβ aggregation in normal aging. While the latter explanation is mechanistically plausible, recent evidence indicates that the seroprevalence of anti-HSV IgG, but not the anti-CMV IgG seroprevalence or anti-CMV IgG levels, doubled the risk of dementia in a cohort of non-demented older adults [45]. On the basis of these results, it does not appear that the pro-inflammatory cytokines-induced production of autoantibodies alone can explain the association between serum levels of anti-HSV IgG and cortical Aβ load in normal aging.

Mounting data indicates that HSV-1 is the human pathogen most frequently linked to Aβ deposition [5, 46], but molecular mechanisms instigating this relationship are not well understood. For instance, acute HSV-1 infection has been shown to alter the APP processing [14] and to increase the enzymes responsible for Aβ formation [47], leading to Aβ accumulation [15]. Increasing research has revealed that Aβ peptides may also have antiviral, antimicrobial and pathogen agglutination properties [48–51], likely due to their common mechanisms in membrane-disruption ability, to their significant role in the biofilm formation sequestering and neutralizing the microbes, and to the extended view that microbial agglutination inhibits microbial entry in cells and facilitates microbial clearance [52]. A chronic activation of these pathways triggered by infections may ultimately exacerbate Aβ deposits, leading to AD progression [53].

The association between anti-HSV IgG levels and cortical Aβ load was restricted to frontal and temporal regions, which is consistent with post-mortem evidence of HSV-1 DNA in Aβ plaques located in temporal and frontal cortices of AD patients and non-demented aged individuals [16]. Together, these results suggest that although Aβ aggregation is likely a programmed immune response to microbial pathogens, the excess buildup of toxic Aβ aggregates results in a chronic inflammatory response that causes AD pathology [5, 54]. Importantly, the relationship between increased risk of dementia and seroprevalence of anti-HSV IgG [45] reinforces the hypothesis that increased levels of anti-HSV IgG in the periphery play a role in dementia development instead of merely reflecting a general augment of blood cells in response to the higher expression of pro-inflammatory cytokines in aging.

Our study further revealed that individuals showing the highest levels of anti-HSV IgG exhibited abnormal patterns of rs-FC with other cortical regions showing early AD pathology, such as the precuneus and temporal cortices [55]. Previous research has shown that HSV-1 binding to cell membranes causes abnormal functional responses in cortical neurons [56, 57]. Thus, HSV-1 infection induced hyperexcitability in cultured rat cortical neurons that increased intracellular Ca2 + signaling, affecting APP processing and ultimately leading to Aβ accumulation [56]. Additionally, HSV-1 infection markedly reduced the expression of synaptic proteins at presynaptic level and impaired synaptic transmission via GSK-3-dependent intraneuronal accumulation of Aβ, which in turn was instigated by intracellular Ca2 + dyshomeostasis [57]. We speculate that this cascade of neuronal events may underlie abnormal regional patterns of rs-FC in individuals showing the highest levels of anti-HSV IgG. However, it remains unclear whether these abnormal patterns of rs-FC represent a compensatory brain response aimed at maintaining the cognitive performance in the absence of obvious signs of neurodegeneration. This alternative hypothesis may account for the lack of relationship between HSV infection and cognitive deficits in the present study.

APOE4, the most common genetic risk factor for developing AD, has been associated with increased susceptibility to HSV-1 infection [20]. Additionally, carriers of the APOE4 genotype who experienced frequent reactivations of HSV-1 had a threefold increased risk of AD [19, 21]. In line with these findings, the present study revealed a moderating role for the APOE4 in the relationship between anti-HSV IgG levels and Aβ load in the anterior cingulate cortex (ACC). The ACC plays a pivotal role in memory, attention and emotions, and it has been considered an integrative hub for decision-making, socially-driven interactions, and empathy-related responses [58, 59]. Evidence suggests that ACC hypometabolism not only is most pronounced in AD patients carrying the APOE4 genotype [60], but it may also predict aging-related cognitive decline [61]. Importantly, the ACC has further shown to accumulate Aβ pathology from early stages of AD [62], which together with HSV-1 infection may boost the excitation/inhibition imbalance of neural circuitries in ACC [63].

This study has some limitations that need to be considered. Although participants were well characterized, the sample size is small and results should be interpreted carefully and replicated with larger cohorts. To minimize this limitation, we only reported results that survived multiple comparisons, showed effect sizes above the minimum established, and were further supported by Bayesian evidence. Moreover, the study sample has twice more females than males, which may impact results given the unquestionable role of gender in the development and outcomes of immune responses [64, 65]. Indeed, 80% of autoimmune diseases occur in women, who typically show stronger immune responses than males. Stronger responses in women produce faster pathogen clearance and better vaccine responsiveness, but they also contribute to increased susceptibility to inflammatory and auto-immune diseases [66]. While statistical analyses were adjusted by sex to minimize gender effects on results, the small sample size impeded us to specifically assess whether sex plays a role in the relationship between HSV infection and Aβ load. Future research should clarify whether the results reported in the present study are restricted to women or occur similarly in both sexes. Given the cross-sectional nature of our study, it remains challenging to establish the prognostic value of these results in AD development. Future longitudinal studies should be carried out to determine to what extent serological evaluation of HSV infection predicts AD progression in cognitively normal older adults. Importantly, increased levels of anti-HSV IgG in serum neither guarantees the occurrence of HSV in the brain nor informs on whether the infection is active or latent. In general, HSV IgM positivity in the periphery is considered a marker of active infection and it probably indicates a recent reactivation. Unfortunately, the small number of positive HSV IgM participants in our sample (*N* = 5) precluded the possibility to study them as a separate group. Finally, associations of HSV with Aβ pathology and/or interactions with APOE4 may have been influenced by HSV-2 infection. However, the overall seroprevalence of HSV-2 in older adults is about 12% [67] and anti-HSV-2 IgG levels have not shown significant interactions with APOE4 genotype [68].

## Data Availability

No datasets were generated or analysed during the current study.

## Abbreviations

AD: Alzheimer’s disease
APOE4: Apolipoprotein E ɛ4 allele
BOLD: Blood-oxygen-level-dependent
CSF: Cerebrospinal fluid
EPI: Single-shot echo-planar
FBB: ^18^F-Florbetaben
GM: Gray matter
HSV-1: Herpes simplex virus type 1
NfL: Neurofilament light chain
rs-FC: Resting state functional connectivity
WM: White matter

## References

[1] Braak H, Braak E (1997). Frequency of stages of Alzheimer-related lesions in different age categories. Neurobiol Aging.

[2] Sojkova J, Zhou Y, An Y, Kraut MA, Ferrucci L, Wong DF (2011). Longitudinal patterns of β-amyloid deposition in nondemented older adults. Arch Neurol.

[3] Musiek ES, Holtzman DM (2015). Three dimensions of the amyloid hypothesis: time, space and ‘wingmen’. Nat Neurosci.

[4] Steel AJ, Eslick GD (2015). Herpes viruses increase the risk of Alzheimer’s disease: a meta-analysis. J Alzheimers Dis.

[5] Itzhaki RF, Lathe R, Balin BJ, Ball MJ, Bearer EL, Braak H (2016). Microbes and Alzheimer’s disease. J Alzheimers Dis.

[6] Warren-Gash C, Forbes HJ, Williamson E, Breuer J, Hayward AC, Mavrodaris A (2019). Human herpesvirus infections and dementia or mild cognitive impairment: a systematic review and meta-analysis. Sci Rep.

[7] Sait A, Angeli C, Doig AJ, Day PJR (2021). Viral involvement in Alzheimer’s disease. ACS Chem Neurosci.

[8] Looker KJ, Magaret AS, May MT, Turner KM, Vickerman P, Gottlieb SL (2015). Global and regional estimates of prevalent and incident herpes simplex virus type 1 infections in 2012. PLoS ONE.

[9] Salinas S, Schiavo G, Kremer EJ (2010). A hitchhiker’s guide to the nervous system: the complex journey of viruses and toxins. Nat Rev Microbiol.

[10] Wilson AC, Mohr I (2012). A cultured affair: HSV latency and reactivation in neurons. Trends Microbiol.

[11] De Chiara G, Piacentini R, Fabiani M, Mastrodonato A, Marcocci ME, Limongi D (2019). Recurrent herpes simplex virus-1 infection induces hallmarks of neurodegeneration and cognitive deficits in mice. PLoS Pathog.

[12] Mangold CA, Szpara ML (2019). Persistent infection with herpes simplex virus 1 and Alzheimer’s disease - A call to study how variability in both virus and host may impact disease. Viruses.

[13] Lövheim H, Gilthorpe J, Adolfsson R, Nilsson LG, Elgh F (2015). Reactivated herpes simplex infection increases the risk of Alzheimer’s disease. Alzheimers Dement.

[14] Shipley SJ, Parkin ET, Itzhaki RF, Dobson CB (2005). Herpes simplex virus interferes with amyloid precursor protein processing. BMC Microbiol.

[15] Wozniak MA, Itzhaki RF, Shipley SJ, Dobson CB (2007). Herpes simplex virus infection causes cellular beta-amyloid accumulation and secretase upregulation. Neurosci Lett.

[16] Wozniak MA, Mee AP, Itzhaki RF (2009). Herpes simplex virus type 1 DNA is located within Alzheimer’s disease amyloid plaques. J Pathol.

[17] Arriagada PV, Growdon JH, Hedley-Whyte ET, Hyman BT (1992). Neurofibrillary tangles but not senile plaques parallel duration and severity of Alzheimer’s disease. Neurology.

[18] Bierer LM, Hof PR, Purohit DP, Carlin L, Schmeidler J, Davis KL (1995). Neocortical neurofibrillary tangles correlate with dementia severity in Alzheimer’s disease. Arch Neurol.

[19] Itzhaki RF, Lin WR, Shang D, Wilcock GK, Faragher B, Jamieson GA (1997). Herpes simplex virus type 1 in brain and risk of Alzheimer’s disease. Lancet.

[20] Burgos JS, Ramirez C, Sastre I, Bullido MJ, Valdivieso F (2003). ApoE4 is more efficient than E3 in brain access by herpes simplex virus type 1. NeuroReport.

[21] Linard M, Letenneur L, Garrigue I, Doize A, Dartigues JF, Helmer C (2020). Interaction between APOE4 and herpes simplex virus type 1 in Alzheimer’s disease. Alzheimers Dement.

[22] Böhm P, Peña-Casanova J, Aguilar M, Hernández G, Sol JM, Blesa R (1998). Clinical validity and utility of the interview for deterioration of daily living in dementia for spanish-speaking communities NORMACODEM Group. Int Psychogeriatr.

[23] Sheikh JL, Yesavage JA (1986). Geriatric Depression Scale (GDS): recent evidence and development of a shorter version. Clin Gerontol.

[24] Gramunt N, Sánchez-Benavides G, Buschke H, Diéguez-Vide F, Peña-Casanova J, Masramon X (2016). The memory binding test: development of two alternate forms into Spanish and Catalan. J Alzheimers Dis.

[25] Alegret M, Valero S, Ortega G, Espinosa A, Sanabria A, Hernández I (2015). Validation of the Spanish version of the Face name associative memory exam (S-FNAME) in cognitively normal older individuals. Arch Clin Neuropsychol.

[26] Miller SA, Dykes DD, Polesky HF (1988). A simple salting out procedure for extracting DNA from human nucleated cells. Nucleic Acids Res.

[27] Greve DN, Salat DH, Bowen SL, Izquierdo-Garcia D, Schultz AP, Catana C (2016). Different partial volume correction methods lead to different conclusions: an (18)F-FDG-PET study of aging. NeuroImage.

[28] Bernal-Rusiel JL, Atienza M, Cantero JL (2008). Detection of focal changes in human cortical thickness: spherical wavelets versus gaussian smoothing. NeuroImage.

[29] Fischl B, Dale AM (2010). Measuring the thickness of the human cerebral cortex from magnetic resonance images. Proc Natl Acad Sci USA.

[30] Glover GH, Li TQ, Ress D (2000). Image-based method for retrospective correction of physiological motion effects in fMRI: RETROICOR. Magn Reson Med.

[31] Yeo IK, Johnson RA (2000). A new family of power transformations to improve normality or symmetry. Biometrika.

[32] Clark TS, Linzer DA (2015). Should I use fixed or random effects?. Pol Sci Res Meth.

[33] Box GEP, Cox DR (1964). An analysis of transformations. J R Stat Soc Ser B.

[34] Bernal-Rusiel JL, Atienza M, Cantero JL (2010). Determining the optimal level of smoothing in cortical thickness analysis: a hierarchical approach based on sequential statistical thresholding. NeuroImage.

[35] Desikan RS, Ségonne F, Fischl B, Quinn BT, Dickerson BC, Blacker D (2006). An automated labeling system for subdividing the human cerebral cortex on MRI scans into gyral based regions of interest. NeuroImage.

[36] Cohen JE (1988). Statistical power analysis for the behavioral sciences.

[37] Liang F, Paulo R, Molina G, Clyde MA, Berger JO (2008). Mixtures of g priors for bayesian variable selection. J Am Stat Assoc.

[38] Lee MD, Wagenmakers EJ. Bayesian cognitive modeling: a practical course. Cambridge University Press; 2013.

[39] Liu H, Qiu K, He Q, Lei Q, Lu W (2019). Mechanisms of blood-brain barrier disruption in herpes simplex encephalitis. J Neuroimmune Pharmacol.

[40] Zhang J, Dai J, Lu Y, Yao Z, O’Brien CA, Murtha JM (2004). In vivo visualization of aging-associated gene transcription: evidence for free radical theory of aging. Exp Gerontol.

[41] Pioli PD, Casero D, Montecino-Rodriguez E, Morrison SL, Dorshkind K (2019). Plasma cells are obligate effectors of enhanced myelopoiesis in aging bone marrow. Immunity.

[42] Ma S, Wang C, Mao X, Hao Y (2019). B cell dysfunction associated with aging and autoimmune diseases. Front Immunol.

[43] Stowe RP, Peek MK, Cutchin MP, Goodwin JS (2012). Reactivation of herpes simplex virus type 1 is associated with cytomegalovirus and age. J Med Virol.

[44] Suzich JB, Cliffe AR (2018). Strength in diversity: understanding the pathways to herpes simplex virus reactivation. Virology.

[45] Vestin E, Boström G, Olsson J, Elgh F, Lind L, Kilander L (2024). Herpes simplex viral infection doubles the risk of dementia in a contemporary cohort of older adults: a prospective study. J Alzheimers Dis.

[46] Itzhaki RF (2021). Overwhelming evidence for a major role for herpes simplex virus type 1 (HSV1) in Alzheimer’s disease (AD); underwhelming evidence against. Vaccines (Basel).

[47] Santana S, Recuero M, Bullido MJ, Valdivieso F, Aldudo J (2012). Herpes simplex virus type I induces the accumulation of intracellular β-amyloid in autophagic compartments and the inhibition of the non-amyloidogenic pathway in human neuroblastoma cells. Neurobiol Aging.

[48] Soscia SJ, Kirby JE, Washicosky KJ, Tucker SM, Ingelsson M, Hyman B (2010). The Alzheimer’s disease-associated amyloid beta-protein is an antimicrobial peptide. PLoS ONE.

[49] Kagan BL, Jang H, Capone R, Teran Arce F, Ramachandran S, Lal R (2012). Antimicrobial properties of amyloid peptides. Mol Pharm.

[50] Kumar DK, Choi SH, Washicosky KJ, Eimer WA, Tucker S, Ghofrani J (2016). Amyloid-β peptide protects against microbial infection in mouse and worm models of Alzheimer’s disease. Sci Transl Med.

[51] Eimer WA, Kumar DK, Shanmugam NK, Rodriguez AS, Mitchell T, Washicosky KJ (2018). Neuron.

[52] Chen D, Liu X, Chen Y, Lin H (2022). Amyloid peptides with antimicrobial and/or microbial agglutination activity. Appl Microbiol Biotechnol.

[53] Moir RD, Lathe R, Tanzi RE (2018). The antimicrobial protection hypothesis of Alzheimer’s disease. Alzheimers Dement.

[54] Jorfi M, Maaser-Hecker A, Tanzi RE (2023). The neuroimmune axis of Alzheimer’s disease. Genome Med.

[55] Insel PS, Mormino EC, Aisen PS, Thompson WK, Donohue MC (2020). Neuroanatomical spread of amyloid β and tau in Alzheimer’s disease: implications for primary prevention. Brain Commun.

[56] Piacentini R, Civitelli L, Ripoli C, Marcocci ME, De Chiara G, Garaci E (2011). HSV-1 promotes Ca2+ -mediated APP phosphorylation and Aβ accumulation in rat cortical neurons. Neurobiol Aging.

[57] Piacentini R, Li Puma DD, Ripoli C, Marcocci ME, De Chiara G, Garaci E (2015). Herpes simplex virus type-1 infection induces synaptic dysfunction in cultured cortical neurons via GSK-3 activation and intraneuronal amyloid-β protein accumulation. Sci Rep.

[58] Bush G, Luu P, Posner MI (2000). Cognitive and emotional influences in anterior cingulate cortex. Trends Cogn Sci.

[59] Lavin C, Melis C, Mikulan E, Gelormini C, Huepe D, Ibañez A (2013). The anterior cingulate cortex: an integrative hub for human socially-driven interactions. Front Neurosci.

[60] Mosconi L, Sorbi S, Nacmias B, De Cristofaro MT, Fayyaz M, Bracco L (2004). Age and ApoE genotype interaction in Alzheimer’s disease: an FDG-PET study. Psychiatry Res.

[61] Pardo JV, Lee JT, Sheikh SA, Surerus-Johnson C, Shah H, Munch KR (2007). Where the brain grows old: decline in anterior cingulate and medial prefrontal function with normal aging. NeuroImage.

[62] Braak H, Braak E (1991). Neuropathological stageing of Alzheimer-related changes. Acta Neuropathol.

[63] Ren SQ, Yao W, Yan JZ, Jin C, Yin JJ, Yuan J (2018). Amyloid β causes excitation/inhibition imbalance through dopamine receptor 1-dependent disruption of fast-spiking GABAergic input in anterior cingulate cortex. Sci Rep.

[64] Forsyth KS, Jiwrajka N, Lovell CD, Toothacre NE, Anguera MC (2024). The conneXion between sex and immune responses. Nat Rev Immunol.

[65] Calabrò A, Accardi G, Aiello A, Caruso C, Candore G (2023). Sex and gender affect immune aging. Front Aging.

[66] Klein SL, Flanagan KL (2016). Sex differences in immune responses. Nat Rev Immunol.

[67] Korr G, Thamm M, Czogiel I, Poethko-Mueller C, Bremer V, Jansen K (2017). Decreasing seroprevalence of herpes simplex virus type 1 and type 2 in Germany leaves many people susceptible to genital infection: time to raise awareness and enhance control. BMC Infect Dis.

[68] Lopatko Lindman K, Weidung B, Olsson J, Josefsson M, Kok E, Johansson A (2019). A genetic signature including apolipoprotein Eε4 potentiates the risk of herpes simplex-associated Alzheimer’s disease. Alzheimers Dement (N Y).

